# Changes in thalamic dopamine innervation in a progressive Parkinson's disease model in monkeys

**DOI:** 10.1002/mds.27921

**Published:** 2019-12-04

**Authors:** Mariana H.G. Monje, Javier Blesa, Miguel Ángel García‐Cabezas, José A. Obeso, Carmen Cavada

**Affiliations:** ^1^ Department of Anatomy, Histology and Neuroscience School of Medicine, Universidad Autónoma de Madrid Madrid Spain; ^2^ HM‐CINAC, HM Puerta del Sur University Hospital Móstoles, and CEU‐San Pablo University Madrid Spain; ^3^ CIBERNED (Center for Networked Biomedical Research on Neurodegenerative Diseases) Instituto Carlos III Madrid Spain; ^4^ Present address: Neural Systems Lab, Department of Health Sciences Boston University Boston Massachusetts USA

**Keywords:** dopamine, macaque monkey, MPTP, Parkinson's disease, thalamus

## Abstract

**Background:**

Dopamine loss beyond the mesostriatal system might be relevant in pathogenic mechanisms and some clinical manifestations in PD. The primate thalamus is densely and heterogeneously innervated with dopaminergic axons, most of which express the dopamine transporter, as does the nigrostriatal system. We hypothesized that dopamine depletion may be present in the thalamus of the parkinsonian brain and set out to ascertain possible regional differences.

**Methods:**

The toxin 1‐methyl‐4‐phenyl‐1,2,3,6‐tetrahydropyridine was administered to adult macaque monkeys using a slow intoxication protocol. The treated macaques were classified into 2 groups according to their motor status: nonsymptomatic and parkinsonian. Dopamine innervation was studied with immunohistochemistry for the dopamine transporter. Topographic maps of the dopamine transporter‐immunoreactive axon distribution were generated and the total length and length density of these axons stereologically estimated using a 3‐dimensional fractionator.

**Results:**

Parkinsonian macaques exhibited lower dopamine transporter‐immunoreactive axon length density than controls in mediodorsal and centromedian‐parafascicular nuclei. Dopamine denervation in the mediodorsal nucleus was already noticeable in nonsymptomatic macaques and was even greater in parkinsonian macaques. Reticular nucleus dopamine transporter‐immunoreactive axon length density presented an inverse pattern, increasing progressively to the maximum density seen in parkinsonian macaques. No changes were observed in ventral thalamic nuclei. Dopamine transporter‐immunoreactive axon maps supported the quantitative findings.

**Conclusions:**

Changes in the dopamine innervation of various thalamic nuclei are heterogeneous and start in the premotor parkinsonian stage. These changes may be involved in some poorly understood nonmotor manifestations of PD. © 2019 The Authors. *Movement Disorders* published by Wiley Periodicals, Inc. on behalf of International Parkinson and Movement Disorder Society.

The primate thalamus is densely innervated by dopaminergic axons.^1^, ^2^ Unlike the rodent thalamus, which is scarcely innervated by dopamine,^3^, ^4^ the primate thalamus has widespread and heterogeneous distribution of dopaminergic axons.^2^, ^5^ Dopamine innervation is densest in the midline nuclei, in some associative nuclei such as mediodorsal (MD) and lateral posterior nuclei, and in the motor thalamus (ventral anterior [VA] and ventral lateral [VL] nuclei). Other nuclei, such as sensory relay nuclei, are weakly innervated.^2^, ^5^ Most thalamic dopaminergic axons express the dopamine transporter (DAT), in particular, those in nuclei beyond the midline, including the MD, ventral nuclei, and the reticular (R) nucleus.^1^, ^2^ The origin of the dopamine innervation of the primate thalamus is complex and diverse. It originates in multiple dopaminergic neuronal populations of the mesencephalon (substantia nigra, ventral tegmental area, and retrorubral area), periaqueductal gray matter, lateral parabrachial nucleus, and hypothalamus.^1^

Dopamine loss in PD has been reported in regions beyond the mesostriatal system, including the globus pallidus, subthalamic nucleus, and motor cortex.^6^ Analyzing changes in dopamine innervation in extrastriatal regions may be relevant to interpreting a series of ill‐understood clinical manifestations in PD patients. These include, for example, attention, visuospatial, and verbal fluency impairment, and abnormal sleep patterns and time estimation deficits.

The thalamus is the only target of basal ganglia outflow to the cortex,^7^ as well as being a significant source of excitatory input to the striatum.^8^, ^9^ Interestingly, the thalamus is one of the few brain regions, besides the substantia nigra, to present early neuronal loss in PD patients and 1‐methyl‐4‐phenyl‐1.2.3.6‐tetrahydropyridine (MPTP)–treated monkeys.^10^, ^11^ Considering the central role of the thalamus in basal ganglia‐cortical interplay and the functional relevance of dopamine in neuromodulation, it is conceivable that changes in thalamic dopamine are involved in the pathophysiology of PD.

Here, we explored the distribution and density of dopamine transporter‐immunoreactive (DAT‐ir) axons in the thalamus of parkinsonian macaques using a model of slow progressive dopamine depletion. DAT is the relevant dopamine marker to be analyzed in the thalamus of parkinsonian macaques: not only is it specific and expressed by the majority of dopaminergic axons in nonmidline nuclei, but the axons expressing DAT are most vulnerable in PD and MPTP‐induced parkinsonism.^12^, ^13^, ^14^ We used unbiased stereological methods to analyze the DAT‐ir axon length density of the following thalamic nuclei: MD, an association nucleus connected to the prefrontal cortex; the ventral nuclei and centromedian‐parafascicular (CnMd‐Pf) complex, which are part of the basal ganglia motor circuitry; and the inhibitory R nucleus, which is in a key position to regulate the thalamus and thalamocortical communication.

## Materials and Methods

### Brain Tissue

The present study used brain tissue from our macaque monkey brain bank. We used tissue from 20 brains of young *Macaca fascicularis* previously studied to stage the degree and extent of the nigrostriatal lesion.^14^, ^15^ The studies were performed according to European and Spanish guidelines (86/609/EEC and 2003/65/EC European Council Directives; and Spanish Government), and were approved by the Committees for Research Ethics of Universities of Navarra and Autónoma de Madrid.

Sixteen macaques aged 4–7 years were treated every 2 weeks with intravenous doses of MPTP (0.5 mg/kg).^14^ Four macaques, which served as controls, only received vehicle injections. The number of MPTP injections varied depending on the systemic response and the motor score reached by each macaque, a consequence of individual differences in susceptibility to the toxin. None of them received l‐dopa or dopamine agonists during the experiment. Animals were evaluated by the research team after each MPTP dose and while the parkinsonian syndrome was progressing until complete stabilization. Motor signs were assessed through the validated Kurlan motor score (range, 0–29 points).^16^

Macaques that exhibited evident parkinsonian signs after the last MPTP injection and that remained affected thereafter until sacrifice were classified as parkinsonian (n = 8; motor score, 9–22). Macaques that maintained normal motor features after the last MPTP dose were considered nonsymptomatic (n = 8); see Supporting Table S1. This group included 4 macaques that developed transient mild parkinsonism and were previously labeled as *recovered*.^14^, ^15^ These macaques exhibited normal motor function for several weeks before sacrifice and were therefore included in the nonsymptomatic group. In sum, the present study allowed us to ascertain the premotor and motor phases of nigrostriatal degeneration mimicking to some extent what occurs in PD.

### Brain Processing and Immunohistochemistry

**Figure 1:**
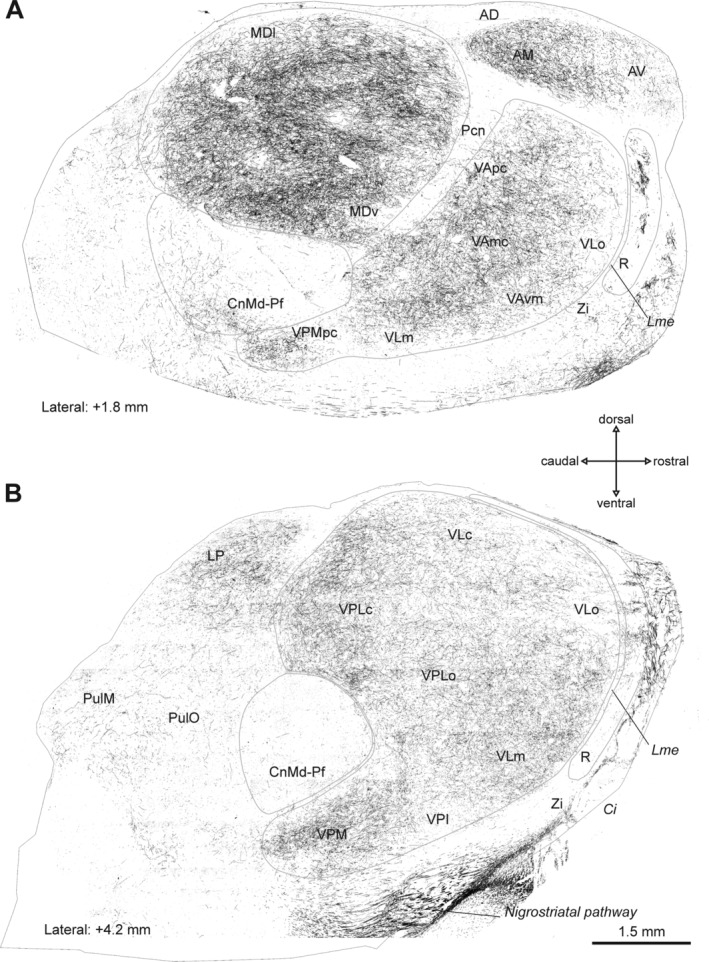
Distribution maps of DAT‐ir axons in the thalamus of a macaque from the control group. Parasagittal sections showing the distribution of DAT‐ir axons in the thalamus of a representative animal of the control group in two lateral planes. Distance from the midline is indicated below each section. Calibration bar applies is the same for both images (**A,B**). Abbreviations: AD, anterodorsal nucleus; AM, anteromedial nucleus; AV, anteroventral nucleus; *Ci*, internal capsule; CnMd‐Pf, centromedian‐parafascicular complex; LP, lateral posterior nucleus; Lme, external medullary lamina; MDl, mediodorsal nucleus‐lateral sector; MDv, mediodorsal nucleus‐ventral sector; Pcn, paracentral nucleus; Pul M, medial pulvinar nucleus; Pul O, oral pulvinar nucleus; R, reticular nucleus; VAmc, ventral anterior nucleus–magnocellular part; VApc, ventral anterior nucleus–parvocellular part; VAvm, ventral anterior nucleus–ventromedial part; VLc, ventral lateral nucleus–caudal part; VLm, ventral lateral nucleus–medial part; VLo, ventral lateral nucleus–oral part; VPI, ventral posterior inferior nucleus; VPLo, ventral posterior lateral nucleus–oral part; VPM, ventral posterior medial nucleus; VPMpc, ventral posterior medial nucleus–parvocellular part; ZI, zona incerta.

**Figure 2:**
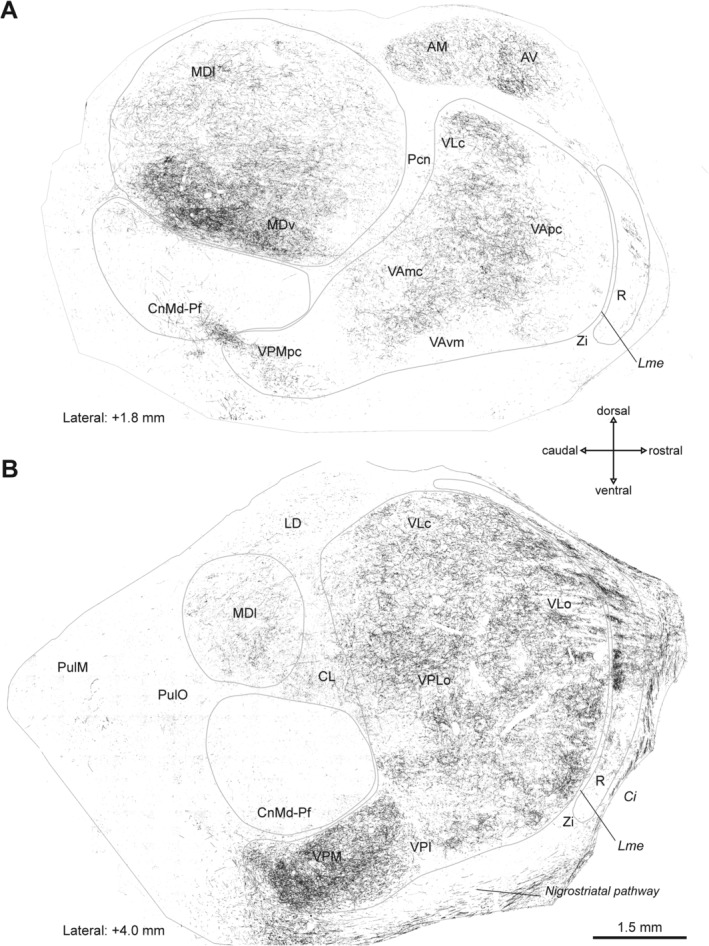
Distribution maps of DAT‐ir axons in the thalamus of a macaque from the nonsymptomatic group. Parasagittal sections showing the distribution of DAT‐ir axons in the thalamus of a representative animal of the nonsymptomatic group in two lateral planes. Differences in DAT‐ir axon density compared wotj the control macaques (Fig. 1) are noticeable, particularly in MD and R. Distance from the midline is indicated below each section. The calibration bar is the same for both images (**A,B**). AM, anteromedial nucleus; AV, anteroventral nucleus; Ci, internal capsule; CL, central lateral nucleus; CnMd‐Pf, centromedian‐parafascicular complex; LD, lateral dorsal nucleus; Lme, external medullary lamina; MDl, mediodorsal nucleus–lateral sector; MDv, mediodorsal nucleus–ventral sector; Pcn, paracentral nucleus; Pul M, medial pulvinar nucleus; Pul O, oral pulvinar nucleus; R, reticular nucleus; VAmc, ventral anterior nucleus–magnocellular part; VApc, ventral anterior nucleus–parvocellular part; VAvm, ventral anterior nucleus–ventromedial part; VLc, ventral lateral nucleus–caudal part; VLo, ventral lateral nucleus–oral part; VPI, ventral posterior inferior nucleus; VPLo, ventral posterior lateral nucleus–oral part; VPM, ventral posterior medial nucleus; VPMpc, ventral posterior medial nucleus–parvocellular part; ZI, zona incerta.

**Figure 3:**
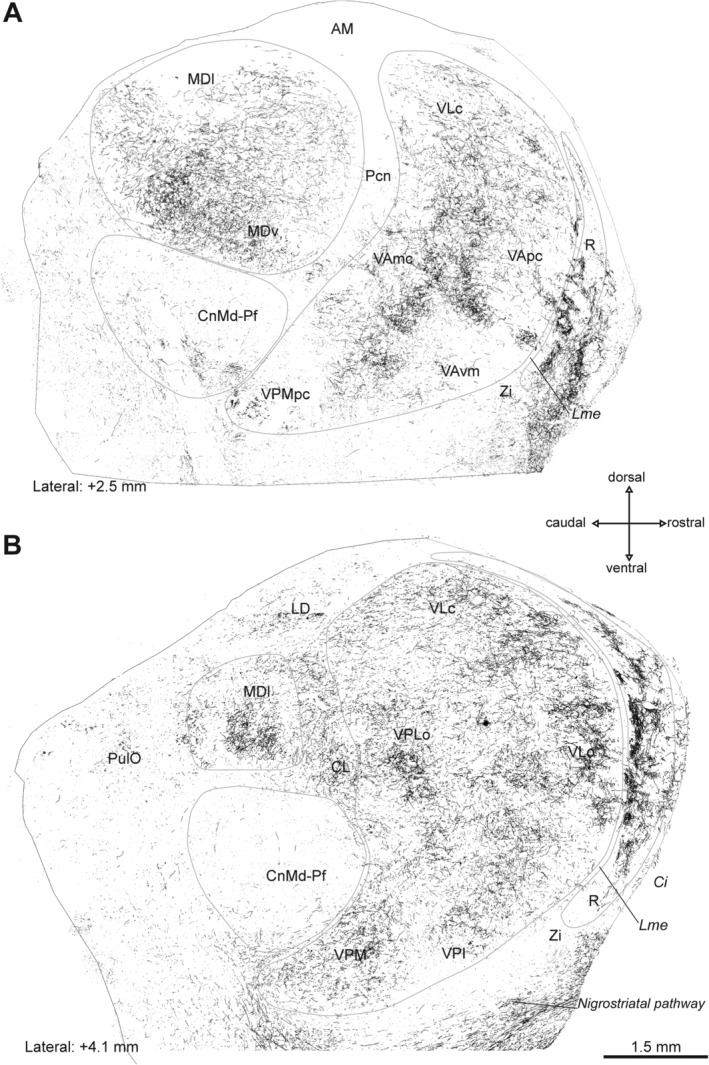
Distribution maps of DAT‐ir axons in the thalamus of a macaque from the parkinsonian group. Parasagittal sections showing the distribution of DAT‐ir axons in the thalamus of a representative animal of the parkinsonian group in two lateral planes. Differences in DAT‐ir axon density compared with the control and the nonsymptomatic macaques (Figs. 1 and 2) are noticeable, particularly in MD and R. Distance from the midline is indicated below each section. The calibration bar is the same for both images (**A,B**). AM, anteromedial nucleus; Ci, internal capsule; CL, central lateral nucleus; CnMd‐Pf, centromedian‐parafascicular complex; LD, lateral dorsal nucleus; Lme, external medullary lamina; MDl, mediodorsal nucleus–lateral sector; MDv, mediodorsal nucleus–ventral sector; Pcn, paracentral nucleus; Pul O, oral pulvinar nucleus; R, reticular nucleus; VAmc, ventral anterior nucleus–magnocellular part; VApc, ventral anterior nucleus–parvocellular part; VAvm, ventral anterior nucleus–ventromedial part; VLc, ventral lateral nucleus–caudal part; VLo, ventral lateral nucleus–oral part; VPI, ventral posterior inferior nucleus; VPLo, ventral posterior lateral nucleus–oral part; VPM, ventral posterior medial nucleus; VPMpc, ventral posterior medial nucleus–parvocellular part; ZI, zona incerta.

See Supporting Appendix 1 for complete information on brain processing. Parasagittal sections were used because they offer more information than coronal sections on the distribution of the dopaminergic innervation of selected macaque thalamic nuclei.^2^, ^5^ In particular, parasagittal sections are relevant for a consistent stereological sampling of DAT‐ir axons in R because DAT innervation in this nucleus is concentrated in its anterior part^2^, ^5^ (Figs. 1, 2, 3). Coronal sections were used for 1 macaque (M17) that had already been prepared using that sectioning plane.^14^, ^15^ Adjacent series of sections were processed to reveal the cytoarchitecture and chemoarchitecture using cresyl violet staining and acetylcholinesterase (AChE) histochemistry, respectively. AChE histochemistry was performed following a protocol described elsewhere.^17^ These series were used to identify the thalamic nuclei and trace their boundaries. Dopamine innervation of the thalamus was studied by immunohistochemistry against DAT in every 20th section containing the thalamus. These sections were stored in a buffered ethylene glycol antifreeze solution until use. For DAT immunohistochemistry we used a primary rat monoclonal antibody (MAB369, 1:1000; Chemicon International, Temecula, CA). The secondary antibody was a polyclonal biotinylated rabbit antirat antiserum (BA4000, 1:400; Vector Laboratories, Burlingame, CA). We followed a previously described protocol using primary antibody incubations of 60 hours at 4°C^2^ with slight modifications in antigen retrieval procedures: the sections were placed in a sodium citrate buffer (pH, 6.00) at constant heat (90°C) in a water bath to cool down for 60 minutes after the sodium citrate treatment.

### Mapping of DAT‐ir Axons in the Thalamus

High‐resolution maps of DAT‐ir axons were generated through a mosaic of 20× magnification pictures taken on a Zeiss Axioskop microscope (Oberkochen, Germany) equipped with a digital camera (DV‐20, MicroBrightField, Europe), using Neurolucida software (MicroBrightField, Colchester, VT). HighPass and Kodalith filters of this software were used to enhance axon‐background contrast (Supporting Fig. S1A–C). Acquisition parameters and filters settings were kept constant for all sections of each macaque brain. The boundaries of the thalamus and other tissue landmarks such as blood vessels were drawn over the immunostained sections and then adjusted onto the adjacent AChE‐stained sections. Here MD, CnMd‐Pf, ventral, and R nuclei were identified, and their borders were traced. The drawings were made on a computer screen using the 4× objective. The cresyl violet–stained sections were used to help to recognize borders between thalamic nuclei. The final maps were prepared using Adobe Photoshop CS5. We analyzed DAT‐ir axon morphologies using 40× and 100× oil immersion objectives. Parcellation of the macaque thalamus followed the terminology and criteria of Olszewski^18^ with some modifications.^17^

### Stereological Quantification of DAT‐ir Axons in the Thalamus

The total length of DAT‐ir axons was estimated by a blinded investigator (M.H.G.M.) using the spaceball stereological approach.^19^, ^20^ We used hemispherical probes combined with unbiased fractionator sampling implemented by StereoInvestigator software (v9.10; MicroBrightField Bioscience, Williston, VT); see Supporting Figure S1D,E. This software controlled the motorized stage of the Zeiss microscope and provided interactive test grids and probes. MD, CnMd‐Pf, ventral, and R thalamic nuclei were analyzed. We used 6 regularly spaced sections covering the mediolateral extent of MD and CnMd‐Pf, and 12 sections for ventral and R nuclei. The hemispherical probes, with a 10‐μm radius, were randomly placed within the section thickness and spaced in sampling grids of 500 × 500 μm (MD and ventral nuclei) and of 200 × 200 μm (CnMd‐Pf and R nuclei). A guard zone 2 μm high was used on top of the sections. After immunohistochemical processing, section thickness ranged between 13 and 15 μm. The 100× oil‐immersion objective was used to identify DAT‐ir axons and their topographical relation with the boundaries of the hemispheres. The precision of the estimates was determined through the coefficient of error (CE) as described for systematic uniform random samples.^21^ CEs obtained ranged between 0.04 and 0.08. DAT‐ir axon length densities, that is, the total length of DAT‐ir axons within the nucleus per volume of the nucleus, were obtained using DAT‐ir axon total length, as estimated by the spaceball approach and the volume of each thalamic nucleus estimated through the Cavalieri method.^21^

### Data and Statistical Analysis

Statistics were performed with SPSS software (v22.0; SPSS Inc., Chicago, IL) and R Studio. Because the data from our experimental groups (n = 4–8) were not normally distributed, all comparisons were made using nonparametric statistical tests (Kruskal‐Wallis test with post hoc analysis using the Mann‐Whitney *U* test). The correlation calculation of DAT‐ir axon length density with earlier data from the same experimental subjects^14^ was performed using the Spearman correlation coefficient. In addition, nonlinear regression was performed. Significance was set at *P* < 0.05. Data from 1 control macaque (M2) and data for the R nucleus from 1 parkinsonian macaque (M17) were excluded because of insufficient tissue sections for optimal stereological study. Outliers were included in the statistical analysis. Mean and standard error the mean are used throughout the text, and median and interquartile range are used in the box‐plot graphic representations.

## Results

### Distribution of DAT‐ir Axons in the Thalamus in Control and MPTP‐Treated Macaques

Figures 1, 2, 3 show maps of DAT‐ir axon distribution in the thalamus of representative cases from the control (Fig. 1), nonsymptomatic (Fig. 2), and parkinsonian (Fig. 3) groups. DAT immunolabeling in the control macaque thalamus showed the previously reported distribution.^2^ MD was densely innervated, as well as some regions of the ventral complex, particularly within VA and VL. DAT immunolabeling in CnMd‐Pf was scarce and mostly located in Pf. In R, DAT‐ir axons predominated in its anterior part (Fig. 1).

**Figure 4:**
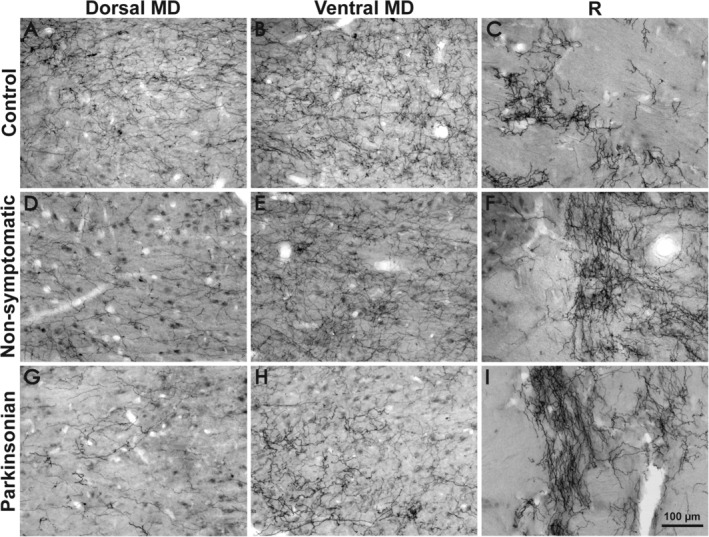
High‐power pictures of DAT‐ir axons in the mediodorsal and reticular nuclei of the control, nonsymptomatic, and parkinsonian groups. Control macaques show dense DAT immunolabeling in the dorsal and ventral regions of the MD nucleus (**A,B**), with scattered axons in the R nucleus (**C**). Nonsymptomatic macaques show a decrease in the DAT‐ir axon distribution in the MD nucleus compared with controls, more noticeable in the dorsal region (**D**) than in the ventral region (**E**). The R nucleus shows an increase in DAT immunolabeling (**F**). Parkinsonian macaques had more considerable depletion in the dorsal and ventral regions of the MD nucleus (**G,H**) compared with the control and nonsymptomatic groups. In the parkinsonian macaques, DAT immunolabeling in the R nucleus (**I**) was markedly increased. Calibration bar in I applies to all images. MD, mediodorsal nucleus; R, reticular nucleus.

MPTP‐treated macaques showed marked changes in DAT immunolabeling in some thalamic nuclei compared with controls, mainly in MD and R (Figs. 2 and 3). DAT‐ir axonal loss was present over the whole anteroposterior axis of MD in both MPTP‐treated groups. In other words, MD showed an early reduction in DAT‐ir axons, already evident in nonsymptomatic macaques (Fig. 2) but more pronounced in parkinsonian macaques (Fig. 3). Dopamine denervation was more noticeable in dorsal MD regions than in ventral regions in both MPTP‐treated groups (Figs. 2, 3, 4D,E,G,H). In CnMd‐Pf, the maps showed no clear differences in DAT‐ir axon distribution (Figs. 1, 2, 3). In the ventral nuclei, DAT‐ir axon density differences between groups were difficult to detect because of the innervation heterogeneity and the difficulty of reliably tracing nuclear borders (Figs. 1, 2, 3). Nevertheless, DAT‐ir axons in the oral part of VL nucleus (VLo) appear denser in MPTP‐treated macaques than in controls (Figs. 1, 2, 3). Both nonsymptomatic and parkinsonian macaques had denser DAT‐ir axon staining in R than controls (Figs. 1, 2, 3, 4C,F,I). This increase in DAT‐ir axons was most evident in the anterior region of the nucleus (Figs. 2, 3). The characteristic patchy appearance of DAT‐ir axons in the R of control macaques was maintained in MPTP‐treated macaques, DAT‐ir axons in R stood out as dense clusters surrounded by zones with lower axonal density (Fig. 4C,F,I).

The fine morphology of DAT‐ir axons across the analyzed thalamic nuclei, including R, was similar to that described previously in non‐MPTP treated macaques^2^: the studied nuclei contained mostly thin varicose fibers with no differences in morphological traits between groups. We did not observe DAT‐ir axons showing increased branching suggestive of sprouting.^22^

### DAT‐ir Axon Length Density in the Thalamus of Control Macaques

**Figure 5:**
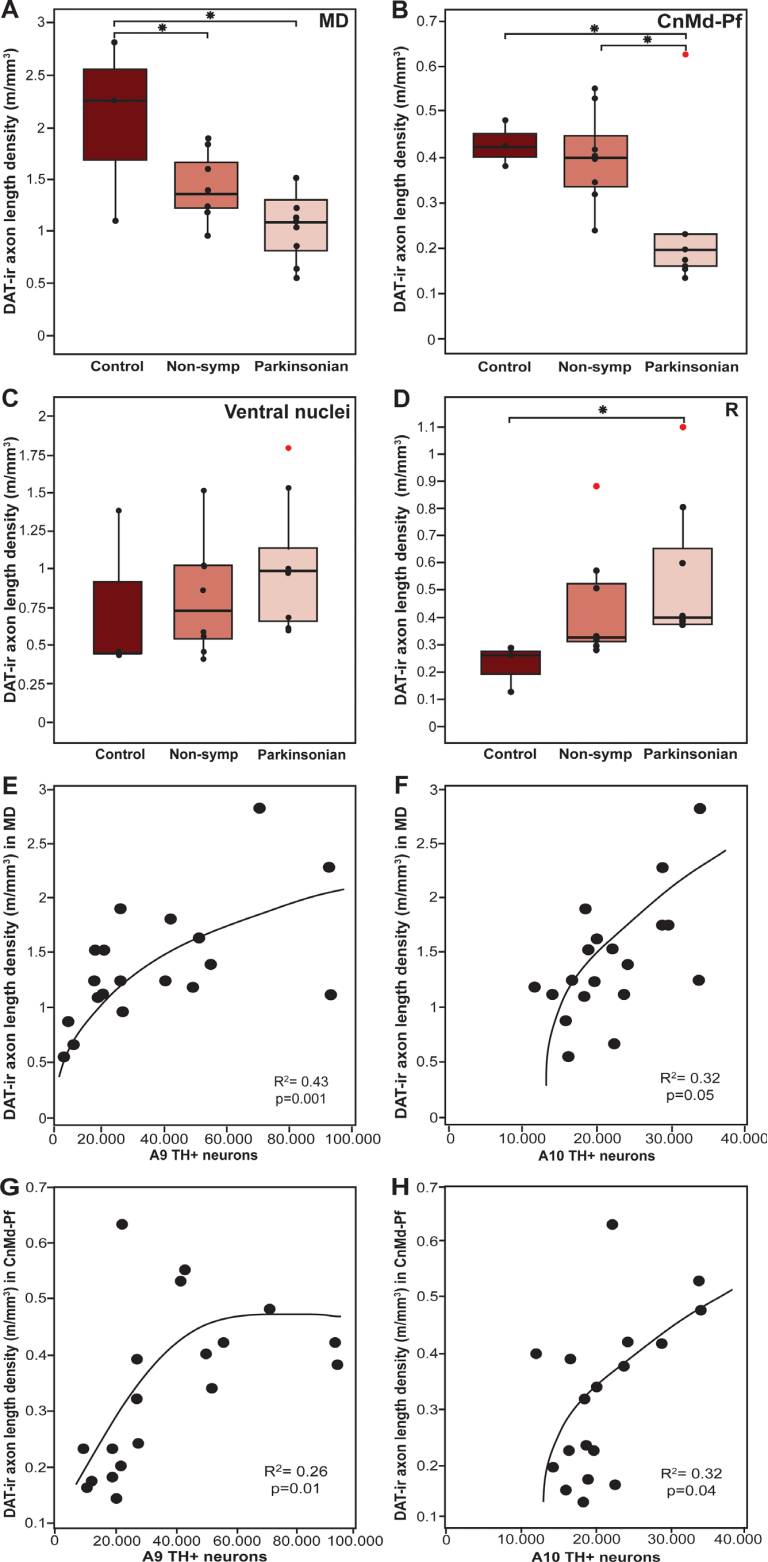
Box‐plot representation of DAT‐ir axon length density and regression analysis between DAT‐ir axon length density and mesencephalic TH+ neurons. DAT‐ir axon length density in the mediodorsal (**A**), centromedian‐parafascicular complex (**B**), and ventral (**C**) and reticular (**D**) nuclei in each animal group is represented. The central horizontal line represents the median value. The bottom and top of the box represent the first and third quartiles. The ends of the whiskers represent the minimum and maximum data values. Dots show each individual value. Outlier data are plotted as individual red points. Significant differences were found between the control group and the nonsymptomatic (*P < 0.05) and parkinsonian (*P < 0.05) groups in the MD nucleus (**A**) and between the parkinsonian group and the control (*P < 0.05) and nonsymptomatic group (*P < 0.05) in the CnMd‐Pf nucleus (**B**). No significant differences were found in the ventral nuclei (**C**). Significant differences were found between the parkinsonian group and the control group in the R nucleus (*P < 0.05) (**D**). Significant polynomial regression was observed between DAT‐ir axon length density in the MD nucleus and TH+ dopaminergic cell loss in A9 (**E**) and in A10 (**F**). Significant polynomial regression was also observed between DAT‐ir axon length density in the CM‐Pf nucleus and TH+ dopaminergic cell loss in A9 (**G**) and in A10 (**H**). [Color figure can be viewed at http://wileyonlinelibrary.com]

DAT‐ir axon length density varied in the different nuclei. The highest length density was present in MD (2.07 ± 0.50 m of DAT‐ir axons/mm^3^) followed by the ventral nuclei (0.76 ± 0.31 m of DAT‐ir axons/mm^3^). CnMd‐Pf and R had low densities of DAT‐ir axons (0.43 ± 0.03 and 0.22 ± 0.05 m/mm^3^, respectively); see Figure 5A–D, control group. These results are consistent with the DAT‐ir axon distribution patterns illustrated in the maps (Fig. 1, Supporting Table S2).

### DAT‐ir Axon Length Density in the Thalamus of MPTP‐Treated Macaques

Thalamic DAT‐ir axons showed varied susceptibility to MPTP in the thalamic nuclei analyzed here. MD and CnMd‐Pf showed decreased DAT‐ir axon length density, whereas R showed increased DAT‐ir axon length density. No differences in DAT‐ir axon length density were detected in ventral nuclei (*P* > 0.05; Fig. 5A–D, Supporting Table S2).

DAT‐ir axon length density in MD was decreased in nonsymptomatic macaques, becoming even lower in parkinsonian macaques. More specifically, compared with controls, DAT‐ir axon length density in MD decreased by 31% in nonsymptomatic macaques (*P* = 0.04) and by 48% in parkinsonian macaques (*P* = 0.04; Fig. 5A). Macaques with more severe parkinsonian signs (ie, Kurlan score ≥18) had greater depletion of DAT‐ir axons (*P* = 0.01). DAT‐ir axon length density in CnMd‐Pf was 43% lower in parkinsonian macaques compared with controls (*P* = 0.04) and 38% lower in parkinsonian macaques compared with nonsymptomatic macaques (*P* = 0.04; Fig. 5B). Thus, DAT‐ir axon length density in the CnMd‐Pf complex did not significantly decrease in the nonsymptomatic macaques but significantly decreased in the parkinsonian macaques relative to both control and nonsymptomatic groups.

In contrast with the above reductions, DAT‐ir axon length density in R increased progressively in MPTP‐treated macaques in parallel with the increased severity of their parkinsonism. Compared with the controls, axon length density increased by 95% in nonsymptomatic macaques and by 145% in parkinsonian macaques (*P* = 0.04; Fig. 5D).

### Associations Between DAT‐ir Axon Length Density in the Thalamus, Motor Status and Mesencephalic TH+ Dopaminergic Neurons

Correlation analyses were performed between DAT‐ir axon length density in the thalamic nuclei, number of mesencephalic dopaminergic neurons, DAT innervation in the striatum, and motor status of the same macaques.^14^ (Supporting Appendix 2 and Fig. S2). In the predictive regression model analyses, MD DAT‐ir axon length density showed a positive nonlinear relationship with TH+ neurons in A9 (*R* ^2^ = 0.43, *P* = 0.001) and A10 (*R* ^2^ = 0.32, *P* = 0.05); see Figure 5E,F). The density of DAT‐ir axons in CnMd‐Pf also showed a positive nonlinear relationship with the number of TH+ neurons in A9 (*R* ^2^ = 0.26; *P* = 0.01) and A10 (*R* ^2^ = 0.32; *P* = 0.04); see Figure 5G,H.

## Discussion

This study provides the first demonstration of widespread changes in the thalamic dopaminergic system in slowly and progressively induced MPTP‐parkinsonism in macaques. We found a decrease in thalamic dopaminergic innervation in MD and CnMd‐Pf that was correlated with the loss of mesencephalic dopaminergic TH+ neurons. We also have shown an unexpected increase in dopaminergic axons in the R nucleus. Changes occurred in all MPTP‐treated macaques, whether nonsymptomatic or symptomatic, but were more overt and intense in the symptomatic parkinsonian macaques.

The stereological method used here allows rigorous quantitative length and volume analyses that do not depend on presumptions about the shape, size, or randomness of the objects under study.^20^ This stereological approach has been previously used to generate unbiased, reliable, and more efficient results than other quantitative analysis methods.^20^, ^23^ The use of parasagittal sections has optimized the stereological study, particularly for estimating DAT‐ir axon length in R, where the anterior portion is the most densely innervated by dopamine. The use of the hemispherical probe for length estimation has the advantage of being isotropic by itself, not requiring orientation in different planes for its proper use, as with virtual planes.^19^, ^24^

### Changes in Dopamine Innervation of Thalamic Nuclei

#### Nuclei showing decreased axon length density

DAT‐ir axon length density declines across MD in nonsymptomatic macaques, and the decline is more pronounced in parkinsonian macaques (Fig. 5A). In addition, these changes paralleled the reduction in TH+ dopaminergic neurons in A9 and, less markedly, in A10 (Fig. 5E,F, Supporting Fig. S2). This contrasts with the notion that the dopamine innervation of MD in macaques arises mainly from TH+ groups A11, A10, and A8 and only to a smaller extent from A9 of the mesencephalon.^1^ The explanation for this contradiction is not apparent. One possibility is that A9 TH+ neurons projecting to MD may exhibit extensive axonal branching, analogous to the large arboreal pattern described for the dopaminergic projection to the rat striatum and thalamus^25^, ^26^; thus, the loss of a few A9 neurons projecting to MD would result in a marked decrease in DAT‐ir axon length density (Fig. 5E,F). This notion is consistent with the early decline, in the nonsymptomatic stage of the model in both MD DAT‐ir axons (Figs. 2, 4D,E, 5A), and A9 TH+ neurons.^14^ In addition, it could also be that the number of A9 neurons projecting to MD is greater than previously recognized.

Regarding the thalamic nuclei integrated in the basal ganglia motor circuitry, only the parkinsonian macaques showed decreased DAT‐ir axon length density in CnMd‐Pf. This decline in DAT‐ir axon length density (Fig. 5B) correlated with the numbers of A9 and A10 TH+ neurons (Fig. 5G,H, Supporting Fig. S2). The ventral nuclei showed no significant changes in DAT‐ir axon length density between groups or any correlation with TH+ neurons (Fig. 5C, Supporting Fig. S2). Thus, the reported functional changes in the motor thalamic nuclei in parkinsonism^27^ should be mainly understood as the consequence of functional circuitry changes in the motor circuit rather than direct loss of dopaminergic afferents to those nuclei. Admittedly, there may be restricted territories within the ventral nuclei undergoing dopamine axonal changes not detected with the stereological approach used here. Our approach demands clear and consistent borders in the regions to be analyzed, which was viable for ventral nuclei taken as a whole, not for individual ventral nuclei.

The decrease of DAT‐ir axon length density in MD and CnMd‐Pf could be because of degeneration of DAT‐ir axons or of phenotype modifications in dysfunctional dopaminergic neurons, as previously suggested for the loss of TH and DAT axons in the striatum of PD patients.^28^

#### Enhanced axon length density in R nucleus

The finding of increased DAT‐ir axon length density in R was unexpected. DAT‐ir axon length density in R does not correlate with the number of TH+ neurons,^14^, ^15^ but does parallel the severity of parkinsonism: the more severe the condition, the higher the DAT‐ir axonal density in the R nucleus (Figs. 2, 3, 4F,I, 5D). To our knowledge, this is the first study describing increased dopamine innervation outside the basal ganglia circuit in the MPTP model.^29^ The net increase in length and density of DAT‐ir axons in R may be because of sprouting, a phenomenon that has been described in the striatum of MPTP models and patients with PD.^22^, ^30^, ^31^, ^32^ However, we did not observe morphological traits suggesting the presence of *new* branches on DAT‐ir axons of the R nucleus in our MPTP‐treated macaques. This is unlike the changes in dopamine axon morphology reported in the striatum of MPTP‐treated monkeys.^22^ Song and Haber (2000) described dopaminergic sprouting in the striatum in monkeys studied at a shorter time after the last MPTP dose than we did here with the thalamus. Perhaps newly formed branches initially display certain specific morphological traits and later adopt the characteristic shape of DAT‐ir axons. Alternatively, a dynamic change in DAT protein expression induced by the parkinsonian changes within the circuit may be the mechanism that underlies the hyperinnervation in R. Indeed, DAT protein membrane expression and trafficking are highly dynamic and depend on neuronal activity.^33^ In any case, be it sprouting or a dynamic change in DAT protein expression, changes in DAT‐ir axonal density in R may be the result of a functional adaptation related to mesostriatal damage, similar to the dopamine hyperinnervation described in the GPi of MPTP macaques.^29^

### Comparison With Previous Studies

Although changes in the dopamine innervation of the thalamus in the parkinsonian brain have never been reported in as much detail as here, our findings concur with previous data in the literature on MPTP monkeys and humans with PD.^34^, ^35^, ^36^, ^37^, ^38^ One study, which used an acute MPTP intoxication protocol (intracarotid and intrastriatal injections) in rhesus monkeys (n = 5), found a qualitative decrease in DAT‐ir axons in the thalamus, including R.^37^ The findings from this study cannot be directly compared with the present ones because of differences in methodology. Our slow MPTP intoxication protocol makes it possible to observe adaptive processes over a fairly long time course in the parkinsonian brain, allowing detection of long‐term variations in DAT‐ir axon density such as the increase in DAT+ fibers in R (Figs. 2, 3, 4, 5A–D).

Biochemical studies using chromatography in the thalamus of MPTP‐treated macaques have provided inconclusive data.^34^, ^35^, ^36^ In MD, Pifl et al described a reduction in dopamine concentration that was already present in asymptomatic stages,^34^, ^35^ although their findings were not replicated.^36^ This later study also analyzed CnMd‐Pf, VA, VL and R, in which no changes in dopamine concentration were detected.^36^ Minimal changes in dopamine concentration might not be possible to detect by chromatography, particularly if the variations are related to the dynamic state of the circuit and not measurable in postmortem studies.^36^ Finally, some neuroimaging studies reported reduced thalamic DAT binding^39^ and D2 dopamine receptors in the medial thalamus of PD patients.^38^ These observations may be in keeping with the reduced dopamine innervation of MD and CnMd‐Pf observed in our study.

### Implications for Circuit Functionality and Relevance to Parkinson's Disease

The dopamine depletion in MD, although widespread, predominantly involves dorsal regions of this nucleus (Figs. 1, 2, 3, 4A,B,D–H). The dorsal MD is extensively connected with the medial and dorsolateral prefrontal cortex and with the anterior cingulate cortex, so dopamine depletion could potentially impair thalamofrontal cortex activity.^40^, ^41^ Patients with MD lesions (ie, vascular, tumoral, etc.) show deficits that are similar to those of patients with frontal cortex damage: inattention, impulsiveness, and working memory defects.^42^ Interestingly, in nonsymptomatic MPTP‐treated monkeys, attention and executive function deficits have been reported,^43^, ^44^ in some way resembling the deficits observed in patients in early PD stages^45^, ^46^, ^47^ and suggestive of dorsolateral prefrontal and anterior cingulate dysfunction. It is reasonable to assume that normal MD activity is necessary for normal prefrontal cortex function^48^ and that dopamine denervation may have an impact on the functions supported by the MD‐frontal circuits.

The CnMd‐Pf complex has been implicated in PD pathophysiology and suggested as a possible target for functional neurosurgery.^49^, ^50^ Neuropathological analyses have shown that neuron loss in CnMd‐Pf is present in nonsymptomatic MPTP macaques and early PD stages and progresses thereafter.^10^, ^11^ The present findings indicate that dopamine denervation is also present in CnMd‐Pf and that it occurs in the symptomatic parkinsonian stage.

Changes in dopamine innervation of R deserve comment. R is an inhibitory nucleus that receives nonreciprocal projections from the cortex and subcortical structures and is reciprocally connected with all thalamic nuclei; each region of R is connected with a set of thalamic nuclei.^51^, ^52^, ^53^ R exerts a modulatory or filtering effect over afferent thalamic activity, making it critical to the generation of thalamocortical rhythms and critically involved in attention and sleep mechanisms.^52^, ^54^ DAT‐ir axons are mostly present in the anterior region of R, and in MPTP‐treated macaques, they increase in the same region (Figs. 1, 2, 3). The R nucleus anterior region receives input from prefrontal and frontal cortical areas^51^, ^53^ and is connected with MD, lateral posterior, VL, and VA nuclei. How the parkinsonism‐associated increase in dopamine affects R inhibitory activity and how this may affect cortico‐thalamo‐cortical dynamics in the parkinsonian brain is currently unknown. Relevantly, dopamine innervation is increased in the two structures that have an inhibitory effect on thalamocortical activity: the R nucleus and the GPi.^29^ In rats, R and Gp receive dopamine innervation from collaterals of the same substantia nigra neurons.^25^, ^55^ Increased dopamine innervation in these two nuclei during parkinsonism may be the result of common mechanisms and may represent a neuroadaptive change with distinct compensatory effects against the massive dopamine denervation occurring in the striatum.

Full understanding of changes in thalamic dopaminergic transmission in PD and PD models requires additional studies on potential changes in dopamine receptors and dopamine synaptic function, as well as on their impact on the thalamocortical circuits involved.

### Conclusions

This study gives evidence of well‐defined and specific changes in thalamic dopamine innervation in nonsymptomatic and symptomatic MPTP‐treated macaques. Interestingly, MD and CnMd‐Pf nuclei sustain marked dopamine denervation, whereas R exhibits hyperinnervation. Thus, the present findings reveal yet another nonstriatal site, the thalamus, in which dopamine changes may affect brain function in the presymptomatic and symptomatic phases of parkinsonism. We speculate that changes in thalamic dopamine innervation may contribute to the pathophysiology of some ill‐understood manifestations of the parkinsonian state such as attention and executive deficits and sleep impairment.

## Authors’ Roles

M.H.G.M.: 1A–C, 2A–C, 3A,B.

J.B.: 1A–C, 2C, 3B.

M.A.G.C.: 1A–C, 2C, 3B.

J.A.O.: 1A,B, 2C, 3B.

C.C.: 1A–C, 2C, 3B.

1) Research project: A. conception, B. organization, C. execution; 2) statistical analysis: A. design, B. execution, C. review and critique; 3) manuscript: A. writing of the first draft, B. review and critique.

## Financial Disclosures of All Authors (for the Preceding 12 Months)

M.H.G.M. is an employee of the Autonomous University of Madrid and HM‐Puerta del Sur University Hospital. J.B. is currently funded by grant S2017/BMD‐3700 (NEUROMETAB‐CM) from Comunidad de Madrid co‐financed with the Structural Funds of the European Union, Fundación BBVA and Fundación Tatiana Pérez de Guzmán el Bueno. M.Á.G.C. is an employee of the Neural Systems Laboratory, Department of Health Sciences, Boston University. J.A.O. is funded from the Ministry of Science and Education of Spain, Fundación La Caixa and CIBERNED; has received honorarium for lecturing in meetings organized by BIAL, Zambon, and Boston Scientific in Spain, as member of Insightec's advisory board, and as member of the Adverse Event Committee for the Biogen randomized immunotherapy trial. C.C. is an employee of the Autonomous University of Madrid and is funded by the Chair UAM‐Fundación Tatiana Pérez de Guzmán el Bueno.

## Supporting information


**TABLE S1.** Macaque monkey features, number of MPTP injections, parkinsonian scores, and survival after MPTP.
**TABLE S2.** Stereological estimations. Total DAT+ axon length, nucleus volume, and DAT+ axon length density of each macaque and nucleus analyzed.
**FIG. S1.** DAT‐ir axon map generation and stereological method. High‐resolution maps of DAT‐ir axons were generated through a mosaic of 20× magnification pictures (A). HighPass (B) and Kodalith (C) filters were used to enhance axon‐background contrast. Stereological images (D,E). The hemispherical probe used as a method of length estimation (D). The gray arrow represents the thickness of the section, the red arrow represents the radius of the hemisphere, and the green arrow, the upper guard area. Axons are depicted in brown (D). Microphotographs of a sampling point (E) from top to bottom. The distance between each image is 1 μm on the *Z* axis, from the top (1) to bottom (14) of the section. The white circles represent consecutive perimeters of the hemisphere. The red arrowhead (8,9) indicates the intersection of a DAT‐ir axon with the hemisphere. The calibration bar applies to all images.
**FIG. S2** Correlation matrix. Correlation matrix between motor scale, DAT‐ir axon length density in mediodorsal (MD DAT+), centromedian‐parafascicular (CnMd‐Pf DAT+), ventral (ventral DAT+),and reticular (R DAT+) nuclei, as well as the dopaminergic innervation of the striatum analyzed by optical density (Str DAT OD) and the numbers of mesencephalic dopaminergic neurons (A8 TH+, A9 TH+, A10 TH+, A11 TH+; Blesa et al, 2012). The color code is on the bar to the right, which gives the correlation scores from ‐1 to 1.
**Appendix 1. Material and Methods. Brain Processing**
After being stable for several weeks in the corresponding motor state, the macaques were deeply anesthetized with intraperitoneal sodium pentobarbital (10 mg/kg). Saline was perfused through the ascending aorta, followed by 4% paraformaldehyde in phosphate buffer (PB) and a series of PB sucrose solutions of increasing concentrations (5%‐10%‐20%). One hemisphere of each brain was stereotaxically blocked in the sagittal plane. Brain blocks were cryoprotected in 30% sucrose for about two weeks under gentle movement at 4° C until sunk. Then, 40 μm parasagittal sections were obtained using a freezing microtome.
**Appendix 2. Results. Correlation analyses**
In MD, DAT‐ir axon length density significantly correlated with motor scale (rho = ‐0.62, *P* = 0.006), optical density in the striatum (rho = 0.77, *P* = 0.005), and number of TH+ neurons in mesencephalic dopaminergic groups A8, A9, and A10 (rho = 0.66, *P* = 0.02; rho = 0.76, *P* = 0.007; rho = 0.59, *P* = 0.01, respectively). DAT‐ir axon length density in the CnMd‐Pf complex correlated with motor scale (rho = ‐0.59, *P* = 0.02), and DAT optical density in the striatum (rho = 0.50, *P* = 0.01). In addition, significant correlations were found between DAT‐ir axon length density in CnMd‐Pf and the numbers of TH+ neurons in A9 and A10 (rho = 0.55, *P* = 0.02; rho = 0.56, *P* = 0.01, respectively), but not with the number of TH+ neurons in A8. In R nucleus, DAT‐ir axon length density did not correlate with motor scale score or DAT optical density in the striatum, but it was correlated with the number of TH+ neurons in group A8 (rho = ‐0.47, *P* = 0.04; Supporting Fig. S2).
