# Supplementary material for: Changes in thalamic dopamine innervation in a progressive Parkinson's disease model in monkeys
Source: Mov Disord. 2019 Dec 4;35(3):419–30. doi: 10.1002/mds.27921 (PMC7154739; doi:10.1002/mds.27921)
Supplement: Supplementary file 1 — TABLE S1. Macaque monkey features, number of MPTP injections, parkinsonian scores, and survival after MPTP. TABLE S2. Stereological estimations. Total DAT+ axon length, nucleus volume, and DAT+ axon length density of each macaque and nucleus analyzed. FIG. S1. DAT‐ir axon map generation and stereological method. High‐resolution maps of DAT‐ir axons were generated through a mosaic of 20× magnification pictures (A). HighPass (B) and Kodalith (C) filters were used to enhance axon‐background contrast. Stereological images (D,E). The hemispherical probe used as a method of length estimation (D). The gray arrow represents the thickness of the section, the red arrow represents the radius of the hemisphere, and the green arrow, the upper guard area. Axons are depicted in brown (D). Microphotographs of a sampling point (E) from top to bottom. The distance between each image is 1 μm on the Z axis, from the top (1) to bottom (14) of the section. The white circles represent consecutive perimeters of the hemisphere. The red arrowhead (8,9) indicates the intersection of a DAT‐ir axon with the hemisphere. The calibration bar applies to all images. FIG. S2 Correlation matrix. Correlation matrix between motor scale, DAT‐ir axon length density in mediodorsal (MD DAT+), centromedian‐parafascicular (CnMd‐Pf DAT+), ventral (ventral DAT+),and reticular (R DAT+) nuclei, as well as the dopaminergic innervation of the striatum analyzed by optical density (Str DAT OD) and the numbers of mesencephalic dopaminergic neurons (A8 TH+, A9 TH+, A10 TH+, A11 TH+; Blesa et al, 2012). The color code is on the bar to the right, which gives the correlation scores from ‐1 to 1. Appendix 1. Material and Methods. Brain Processing After being stable for several weeks in the corresponding motor state, the macaques were deeply anesthetized with intraperitoneal sodium pentobarbital (10 mg/kg). Saline was perfused through the ascending aorta, followed by 4% paraformaldehyde in phosphate buffer (PB) and a series [file MDS-35-419-s001.docx]

**Supporting Information**

| **Table S1.** Macaque monkey features. number of MPTP injections. parkinsonian scores and survival after MPTP. | | | | | | |
| --- | --- | --- | --- | --- | --- | --- |
| **Group** | **Age (y/o)** | **Weight (Kg)** | **Number of MPTP injections** | **Kurlan Score after MPTP^a^** | **Kurlan Score before sacrifice^b^** | **Survival time (days)^c^** |
| Controls (n=4) | 5.8±0.75 | 4.9±1.18 | N/A | N/A | N/A | N/A |
| Non-symptomatic (n=8) | 4.63±0.24 | 4.35±0.48 | 2.25±0.41 | 4.55±1.22 | 0±0 | 89.4±26.1 |
| Parkinsonian (n=8) | 5.65±0.85 | 3.6±0.26 | 6.13±1.44 | 21.0±1.76 | 15.38±2.05 | 91.9±17.5 |
| Data show mean±SEM   1. Scores one week after the last MPTP injection 2. Scores one week before sacrifice 3. Survival time after last MPTP injection | | | | | | |

**Table S1**

**Table S2**

**Table S2. Stereological estimations**

| Macaque | Group | MD | | | CnMd-Pf | | | Ventral nuclei | | | R | | |
| --- | --- | --- | --- | --- | --- | --- | --- | --- | --- | --- | --- | --- | --- |
|  |  | Total length of DAT+ axons (m) | Nucleus volume  (mm^3^) | Length density of DAT+ axons (m/mm^3^) | Total length of DAT+ axons (m) | Nucleus volume  (mm^3^) | Length density of DAT+ axons (m/mm^3^) | Total length of DAT+ axons (m) | Nucleus volume  (mm^3^) | Length density of DAT+ axons (m/mm^3^) | Total length of DAT+ axons (m) | Nucleus volume  (mm^3^) | Length density of DAT+ axons (m/mm^3^) |
| M1 | Control | 126.91 | 45.00 | 2.82 | 9.66 | 20.06 | 0.48 | 177.29 | 128.20 | 1.38 | 16.61 | 56.64 | 0.29 |
| M2 | Control | 22.47 | 25.00 | 0.90 | 5.06 | 14.18 | 0.36 | 38.44 | 116.00 | 0.33 | 7.07 | 33.50 | 0.21 |
| M3 | Control | 92.43 | 40.80 | 2.27 | 8.65 | 20.67 | 0.42 | 70.17 | 151.80 | 0.46 | 6.28 | 50.66 | 0.12 |
| M4 | Control | 53.89 | 48.40 | 1.11 | 7.23 | 19.26 | 0.38 | 71.95 | 161.20 | 0.45 | 11.75 | 46.05 | 0.26 |
| M5 | Non-symp | 70.99 | 43.60 | 1.63 | 8.82 | 25.60 | 0.34 | 251.29 | 165.0 | 1.52 | 42.51 | 47.62 | 0.89 |
| M6 | Non-symp | 65.96 | 53.20 | 1.24 | 15.22 | 28.61 | 0.53 | 91.96 | 162.40 | 0.57 | 28.53 | 55.87 | 0.51 |
| M7 | Non-symp | 62.03 | 44.60 | 1.39 | 9.10 | 21.89 | 0.42 | 103.05 | 118.20 | 0.87 | 15.62 | 49.09 | 0.32 |
| M8 | Non-symp | 53.23 | 45.00 | 1.18 | 9.00 | 22.30 | 0.40 | 61.86 | 131.00 | 0.47 | 11.42 | 35.74 | 0.32 |
| M9 | Non-symp | 108.70 | 60.00 | 1.81 | 10.84 | 19.74 | 0.55 | 158.17 | 150.60 | 1.05 | 17.35 | 52.13 | 0.33 |
| M10 | Non-symp | 73.67 | 38.80 | 1.90 | 7.42 | 23.49 | 0.32 | 148.58 | 145.60 | 1.02 | 29.21 | 52.48 | 0.56 |
| M11 | Non-symp | 57.42 | 46.40 | 1.24 | 10.60 | 27.10 | 0.39 | 65.30 | 154.40 | 0.42 | 14.45 | 50.94 | 0.28 |
| M12 | Non-symp | 57.91 | 60.20 | 0.96 | 5.67 | 23.33 | 0.24 | 92.49 | 156.80 | 0.59 | 19.62 | 66.72 | 0.29 |
| M13 | Park | 65.15 | 43.00 | 1.52 | 4.60 | 25.54 | 0.18 | 134.61 | 134.40 | 1.00 | 20.31 | 54.05 | 0.38 |
| M14 | Park | 54.62 | 48.80 | 1.12 | 4.83 | 23.58 | 0.20 | 91.21 | 151.20 | 0.60 | 18.82 | 50.59 | 0.37 |
| M15 | Park | 66.53 | 43.80 | 1.52 | 11.82 | 18.75 | 0.63 | 264.87 | 148.80 | 1.78 | 55.74 | 50.43 | 1.11 |
| M16 | Park | 55.51 | 51.00 | 1.09 | 2.99 | 21.15 | 0.14 | 143.04 | 143.00 | 1.00 | 36.44 | 44.77 | 0.81 |
| M17 | Park | 26.27 | 20.60 | 1.24 | 2.52 | 10.91 | 0.23 | 126.34 | 81.40 | 1.55 |  |  |  |
| M18 | Park | 37.44 | 43.00 | 0.87 | 3.22 | 19.90 | 0.16 | 149.84 | 153.40 | 0.98 | 37.06 | 62.27 | 0.60 |
| M19 | Park | 37.49 | 56.40 | 0.66 | 3.96 | 23.33 | 0.17 | 124.27 | 181.80 | 0.68 | 28.19 | 69.02 | 0.41 |
| M20 | Park | 19.32 | 35.00 | 0.55 | 4.55 | 19.81 | 0.23 | 75.83 | 123.60 | 0.61 | 22.88 | 61.31 | 0.37 |

Total DAT+ axon length, nucleus volumen and DAT+ axon length density of each macaque and nucleus analyzed are shown. Abbreviations: MD. mediodorsal nucleus; CnMdPf. centromedian-parafascicular; Non-symp. Non symptomatic; Park. Parkinsonian; R. reticular nucleus

**Figure S1**

| 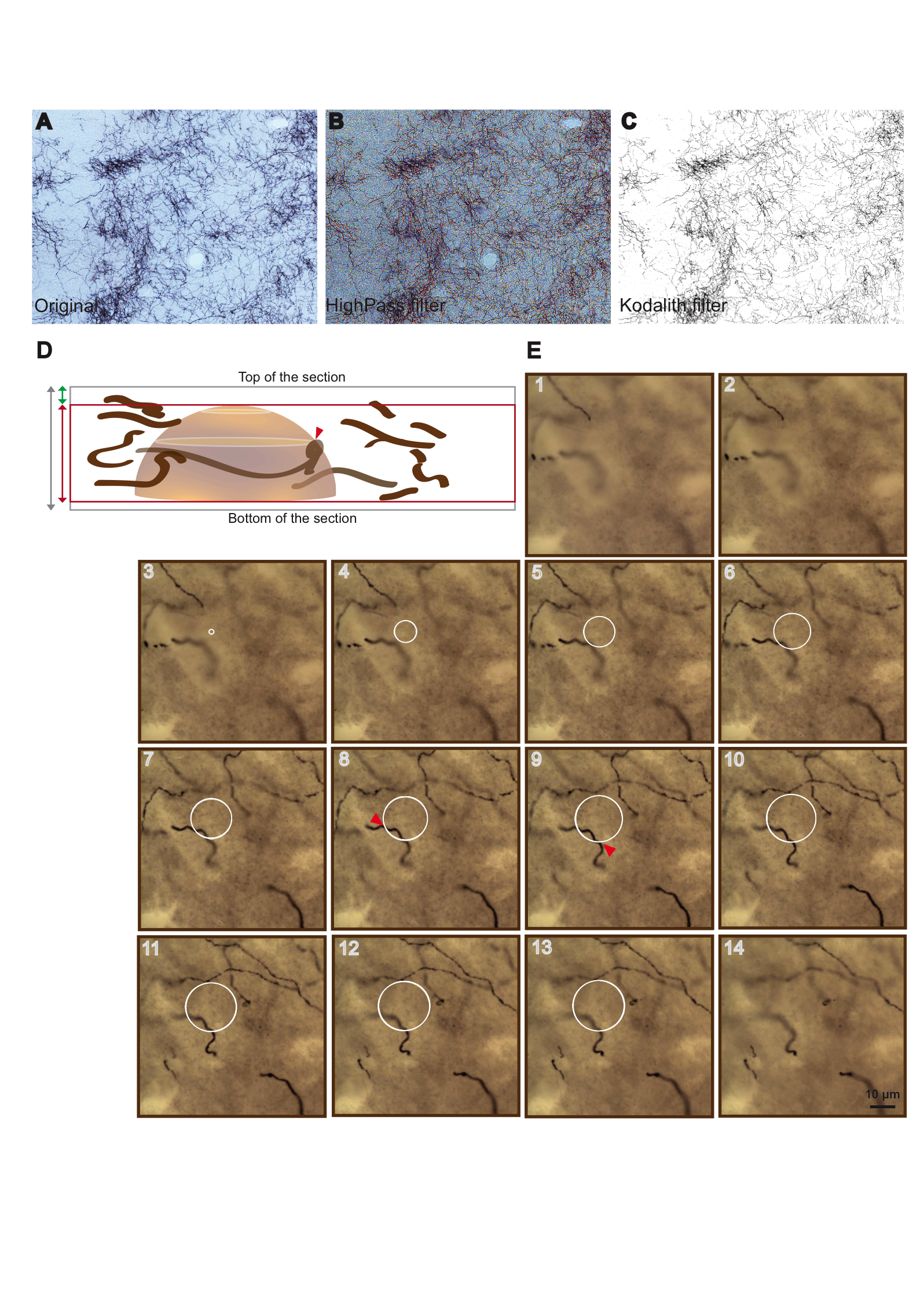 |
| --- |
| **Figure S1. DAT-ir axon map generation and stereological method.**  High-resolution maps of DAT-ir axons were generated through a mosaic of 20x magnification pictures (A). HighPass (B). and Kodalith (C) filters were used to enhance axon-background contrast. Stereological images (D.E). The hemispherical probe used as a method of length estimation (D). The grey arrow represents the thickness of the section. the red arrow represents the radius of the hemisphere and the green arrow. the upper guard area. Axons are depicted in brown (D). Microphotographs of a sampling point (E). from top to bottom. The distance between each image is 1 μm on the Z-axis. from the top (1) to bottom (14) of the section. The white circles represent consecutive perimeters of the hemisphere. The red arrowhead (8,9) indicates the intersection of a DAT-ir axon with the hemisphere. Calibration bar applies to all images. |

**Figure S2**

|  |
| --- |
| **Figure S2. Correlation matrix.** Correlation matrix between motor scale. DAT-ir axon length density in mediodorsal (MD DAT+). centromedian-parafascicular (CnMd-Pf DAT+). ventral (Ventrals DAT+) and reticular (R DAT+) nuclei, as well as the dopaminergic innervation of the striatum (Str DAT OD) and the numbers of mesencephalic dopaminergic neurons (A8 TH+. A9 TH+. A10 TH+. A11 TH+) (Blesa et al.2012). The color code is on the bar to the right; which gives the correlation scores from -1 to 1. |

**Appendix 1**

**Appendix 1. Material and methods. Brain processing**

After being stable for several weeks in the corresponding motor state, the macaques were deeply anesthetized with intraperitoneal sodium pentobarbital (10 mg/kg). Saline was perfused through the ascending aorta, followed by 4% paraformaldehyde in phosphate buffer (PB) and a series of PB sucrose solutions of increasing concentrations (5%-10%-20%). One hemisphere of each brain was stereotaxically blocked in the sagittal plane. Brain blocks were cryoprotected in 30% sucrose for about two weeks under gentle movement at 4º C until sunk. Then, 40 μm parasagittal sections were obtained using a freezing microtome.

**Appendix 2. Results. Correlation analyses**

In MD. DAT-ir axon length density significantly correlated with motor scale (rho= -0.62, p= 0.006). optical density in the striatum (rho= 0.77, p= 0.005), as well as with the number of TH+ neurons in mesencephalic dopaminergic groups A8. A9 and A10 (rho= 0.66, p= 0.02; rho= 0.76, p= 0.007; rho= 0.59, p= 0.01. respectively). DAT-ir axon length density in the CnMd-Pf complex correlated with motor scale (rho= -0.59, p= 0.02), and DAT optical density in the striatum (rho= 0.50, p= 0.01). In addition. significant correlations were found between DAT-ir axon length density in CnMd-Pf and the numbers of TH+ neurons in A9 and A10 (rho= 0.55, p= 0.02; rho= 0.56, p= 0.01. respectively). but not with the number of TH+ neurons in A8. In the R nucleus DAT-ir axon length density did not correlate with motor scale score or DAT optical density in the striatum, but it was correlated with the number of TH+ neurons in group A8 (rho= - 0.47. p= 0.04). (Supporting Fig. S2).
